# Micropreconcentrators: Recent Progress in Designs and Applications

**DOI:** 10.3390/s22041327

**Published:** 2022-02-09

**Authors:** Agnieszka Stolarczyk, Tomasz Jarosz

**Affiliations:** Department of Physical Chemistry and Technology of Polymers, Silesian University of Technology, 44-100 Gliwice, Poland; agnieszka.stolarczyk@polsl.pl

**Keywords:** preconcentrator, volatile organic compound, VOC, exhaled breath, MEMS

## Abstract

The detection of chemicals is a fundamental issue of modern civilisation, however existing methods do not always achieve the desired sensitivity. Preconcentrators, which are devices that allow increasing the concentration of the intended analyte via e.g., adsorption/desorption, are one of the solutions for increasing the sensitivity of chemical detection. The increased detection sensitivity granted by preconcentration can be used to miniaturise detection instruments, granting them portability. The primary goal of this review is to report on and briefly explain the most relevant recent developments related to the design and applications of preconcentrators. The key design elements of preconcentrators and the emerging area of liquid-phase preconcentrators are briefly discussed, with the most significant applications of these devices being highlighted.

## 1. Introduction

The ability to qualitatively and quantitatively detect the presence of chemicals is one of the foundations of modern society. Chemical sensing and detection methods have found countless applications, with monitoring of technological processes [1,2], controlling product quality [3], monitoring the environment [4], diagnostics and health management [5], as well as constituting an element of early warning systems [6] being the most significant fields of their use. Although numerous parameters are used to describe the performance of the multitude of existing detection methods, the key parameter for most applications is the limit of detection (LoD), as it describes the lowest concentration of the intended analyte that can be observed via the selected detection method.

Depending on the specific field and area of application, the employed definitions of LoD may vary significantly [7], however, regardless of employed definition, the issue of insufficient sensitivity of some detection methods persists and can be encountered in a broad variety of applications. This is exemplified by the issues of the on-road monitoring of NO_X_ [8], the measurement of thyroglobulin in human serum [9], and detecting contaminants, such as volatile organic compounds (VOCs) [10] and mercury cations [11].

The issue of insufficient sensitivity of detection methods can in many cases be solved directly, by either improving the utilised detection method or developing a more sensitive, analyte-specific detection method, as seen in the above examples. In many cases, however, a sufficiently sensitive method may not be available, may be prohibitively expensive, or, while being sufficiently sensitive, may suffer from the presence of interfering agents present in the intended sample. One of the solutions to such cases is to employ preconcentration methods.

IUPAC defines preconcentration as: “An operation (process) as the result of which microcomponents are transferred from the sample of larger mass into the sample of smaller mass, so that the concentration of the microcomponents is increased. Examples include the decrease in solvent volume during distillation or evaporation and the transfer of microcomponents from an aqueous solution into a smaller volume of organic solvent by extraction” [12].

The majority of preconcetrators utilises adsorption/desorption phenomena, due to the fact that they are primarily designed for increasing the concentration of gases and vapours (e.g., VOCs). Consequently, the typical operating cycle of this type of preconcentrator can be summarised as:

(1) Adsorption of gas or vapour molecules at room temperature;
(2) Desorption of the molecules at a higher temperature and their injection into the sampling unit of a coupled detection method;
(3) Cooling the preconcentrator down to room temperature before the next sampling and analysis.

This type of preconcentrators is typically coupled with gas chromatography (GC) as the detection method. The popularity of coupling preconcentration with GC stems from the fact that it allows the GC instrument to be miniaturised, simultaneously reducing its cost and increasing its portability, allowing field use of this method. Preconcentration is also coupled with other detection methods (e.g., metal oxide sensors), in which case its use is primarily to amplify the sensitivity of those detection methods, particularly for analytes exhibiting relatively low volatility.

Other types of preconcentrators, operating for analytes in the liquid phase are also described in the literature, becoming an emergent solution due to the rapid progress in the field of micro-electro-mechanical systems (MEMS). Such preconcentrators typically utilise e.g., electric field gradients rather than adsorption/desorption phenomena to achieve preconcentration and are discussed separately.

Numerous designs of preconcentrators have been reported to date, differing in terms of phenomenon utilised for preconcentration, device size, shape, and volume of the preconcentration cavity, as well as the implementation of the mechanism for releasing the concentrated analyte from the device. Both types of preconcentrators have been fabricated using a variety of methods from a multitude of materials, with gas-phase preconcentrators also utilising numerous types of adsorbents. This variation is further increased due to the preconcentrators being coupled with a range of detection methods.

In light of the above, a direct comparison and evaluation of the performance of each device is not feasible and the information we provide about the achieved preconcentration factor or LoD should always be taken in context of the method employed for detecting the preconcentrated analyte. Consequently, the aim of this work is to serve as a guide to the recent (approx. 3 years) developments in the design and applications of preconcentrators and to highlight the most relevant advantages and drawbacks of the reported devices and applications.

## 2. Literature Review

Among recent literature dedicated to preconcentrators, reports focus on all aspects of the design of preconcentrators and their applications. The traditional approach to preconcentration, relying on the adsorption of the analyte and its thermally-induced desorption, remains extremely popular. Nevertheless, great research interest has been recently devoted to exploring other phenomena and methods, such as microextraction and electrokinetic trapping, for the purpose of preconcentration.

In terms of applications, the traditional adsorption/desorption-based micropreconcentrators (μPCs) have been primarily studied with the detection of volatile organic compounds (VOCs) in mind. In this aspect, two main groups of μPC applications are involved in augmenting the detection of environmental contaminants and in personalised medical diagnostics, with the latter use of μPCs assisting the monitoring of the presence and concentrations of various VOCs in exhaled breath. A relevant area of traditional μPC application is the detection of gaseous species (e.g., ethylene) and volatile inorganic susbtances (e.g., mercury vapours), even if it is less represented among the most recent works. Conversely, the applications of μPCs utilising e.g., microextraction, are much more varied and include the detection of acidic drugs (e.g., ibuprofen), heavy metal ions (e.g., Hg^2+^), and biomolecules (e.g., prostate-specific antigen).

### 2.1. Preconcentrators Utilising Adsorption and Desorption

Preconcentrators relying on adsorption/desorption phenomena are currently the most commonly used and reported in the literature of type of devices. The design features, such as the shape of the preconcentration chamber and existence and features of a planar or three-dimensional microstructure within the preconcentration chamber of the μPC directly effect the attainable performance benchmarks of the device [13]. Nevertheless, the influence of the adsorbent utilised in the μPC should also not be underestimated, as it can drastically affect the performance of a device based on a given design [14]. These two aspects, along with issues arising from their superposition (e.g., the use of a “loose” adsorbent as opposed to the deposition of an adsorbent layer onto the interior of the preconcentration chamber) constitute a broad array of factors relevant to the performance of adsorption/desorption-based μPCs. In the following subsections, the various approaches to the development of this type of μPCs are discussed, highlighting the relevant design and material choices where relevant.

#### 2.1.1. Preconcentrators for VOC Contaminants

The idea of coupling a passive diffusion-based preconcentrator with an injector device has been recently proposed, with the coupled device being intended for the μ-GC analysis of volatile organic compounds (VOCs) [15]. Although it contains fairly standard materials (e.g., use of Carbopack brand adsorbents), the system maintained a constant sampling rate in a wide range of xylene concentrations (8–5600 mg/m^3^), achieving high desorption efficiencies and making the system an interesting sampling solution. The presented idea of a passive PC is beneficial when considered in terms of power consumption. However, such a solution is expected to result in issues at the stage of thermal desorption, because the temperature must be high enough to overcome the heat of (vapour) adsorption and the transfer rate must be sufficient to overcome the back-diffusion caused by the heating process to avoid vapour loss back through the inlet opening. Such phenomena are expected to deteriorate the dynamics of the release of the analyte towards the detector.

A very simple to build and cheap micropreconcentrator (μPC) dedicated to detecting air contamination with the BTEX (Benzene, Toluene, Ethylbenzene, and Xylenes) group of VOCs, paired with a GC unit was reported. The μPC was a stainless steel tube filled with an adsorbent, which was the commercially available activated carbon, Carbopack B (SUPELCO, Bellefonte, PA, USA). Rapid heat exchange was provided by an aluminium block equipped with ceramic heaters through which the tube passed. That system was able to preconcentrate BTEX enough to reduce the limits of detection down to 0.057, 0.150, 0.368, 0.396, and 0.418 ppb for benzene, toluene, ethylbenzene, m/p-xylenes, and o-xylene, respectively [16]. The proposed solution has one significant drawback, that is high thermal mass. Despite the relatively high power consumption of the device (up to 70 W), a limited range of heating rates (0.25–5.5 ${}^{\circ }$C/s) was achieved. Although the limited heating rate, which is crucial for inducing the rapid desorption of the analyte and its injection into the sampling unit, did not adversely affect the GC results, it significantly limits the portability and autonomy of the system. This is especially so when compared with e.g., MEMS-based μPCs that achieve heating rates of up to 314 ${}^{\circ }$C/s, while requiring less power (up to 16 W).

A worthwhile effort in terms of developments relevant to the employed adsorbent is described in the report dedicated to the use of a metal organic framework embedded metal foam (MOFM) adsorbent. The metal oxide framework (MOF-5) embedded in this foam is made of octahedral Zn–O–C clusters and benzene links, which are exceptionally rigid and highly porous, making MOF-5 as an effective adsorbent material for sampling and trapping BTEX. The μPC consists primarily of the adsorption chamber fabricated from silicon into a MEMS, microheaters, and resistive temperature sensor, which acts as a trigger for a GC equipped with a FID detector. The measuring equipment used did not allow for the separation of the individual BTEX components. In the study, the authors examined the total BTEX content and the device detected the analyte content at the level of 100 ppb and obtained a preconcentration factor of 144. An analogous μPC was also made using commercially available RAD145 and Carbotrap B adsorbents, achieving much worse operating parameters of such devices, respectively 55 and 36. The undoubted advantage of MOFM-packed μPC is its low desorption temperature, high thermal conductivity, high specific surface area, and low pressure drop. In particular, the low pressure drop characteristic allows the preconcentration device to be miniaturised, which is advantageous for portable applications. Perhaps a change in the construction of the μPC or the detector will allow for better LoD limits. It seems particularly important to investigate the sorption selectivity of individual BTEX components [17].

It was also found that nanoporous silica material adsorbs VOC particles using a commercial total VOC photoionization detector. The authors showed that VOCs are desorbed at different temperatures, depending on their boiling point and affinity to the porous surface. VOC adsorption is proportional to the concentration of VOC in the environment and is fully reversible. Measurements were conducted for six different VOCs (Benzene, Toluene, Xylenes, Limonene, and MEK), demonstrating that a fused silica preconcentrator has the potential to discriminate between VOCs [18]. In this work, the authors proposed an unconventional approach to the idea of μPCs, as their efforts were focused on providing the possibility of controlled desorption of individual analytes by using a slower rate ramp at the expense of sensitivity of μPC itself (lower desorption peak). Therefore, the proposed μPC may perform two functions simultaneously.

A tubular preconcentrator for formaldehyde detection using portable 2-dimensional gas chromatography equipped with photoionization detectors is presented in [19]. The preconcentrator consisted of a deactivated fused silica tube (0.53 mm id and 0.69 mm od) filled with three segments of absorption materials (i.e., Carbopack™ B, Carbopack™ X, and Carboxen^®^ 1000 with a weight of 1 mg each) and sealed with a segment of copper wire on each end. This system allows repeatable formaldehyde detection with a detection limit (at 3σ) of 0.23 ppb (*V*/*V*) with only 6 min of sampling time. Simultaneous analysis of formaldehyde and BTEX were 8 ppb, 51 ppb, 70 ppb, 75 ppb, and 75 ppb, respectively. That said, although very high sensitivity and resolution were achieved, the latter especially is likely to originate from the use of a 2-dimensional GC device used in the manufactured device rather than from the μPC itself.

μPC chips made of borosilicate glass containing an integrated heater and RTD elements were developed in a simple-to-manufacture microfabricated system to be used with MEMS-based chemical sensing applications. Cavities and microfluidic channels were created using a wet etch process with hydrofluoric acid, portions of which can be performed outside of a cleanroom, instead of the more common deep reactive ion etch process. The integrated heater and resistance temperature detectors (RTDs) were created with a photolithography-free technique enabled by laser etching. The μPC chips are 2.54 cm on each side and 0.14 mm thick. The sorbent bed located at the center of the chips holds 6.994±0.821 mg of Tenax TA sorbent, which is a sorbent designed specifically for trapping volatiles and semi-volatiles from air [20]. The chips have the capability of detecting 4-ethyltoluene, benzyl chloride, and 2-hexanone concentrations as low as 22 ppb with a sampling time as low as 2 min with GC-FID detection. The same chip was used in the authors’ later work to produce a portable and wearable sampler that collects environmental VOCs in a person’s immediate “exposure envelope” system and also records ambient temperature, humidity, and location (via GPS) during sampling, and the chip cartridges can be used in sequence over time to complete a profile of individual chemical exposure over the course of hours/days/weeks/months [21].

Kuo et al. proposed MEMS μPC employing a carbon molecular sieve membrane for the detection of ethanol, isopropanol, and acetone. A carbon membrane was injected by carbonization of poly(vinylidene chloride) directly in the channel of the preconcentrator chamber. The layering was monitored by SEM imaging and the amount of adsorbent in the chip was 1.4 mg. The membrane area determined by the BET method was 899 m^2^/g and is similar to commercially available carbon adsorbents. GC-FID was used as the detector. Detection limits in terms of concentration using 1 L as the gas sample volume are 2.3 ppb ethanol, 2.0 ppb acetone, 1.3 ppb ethyl acetate, and 0.4 ppb benzene. A significant disadvantage of CMSM is the difficult desorption of BTEX [22].

A metal-organic framework, composed of Cu^2+^ cations and 1,3,5-benzenetricarboxylate anions was used as an active layer in a microfluidic nerve agent simulant (dimethyl methylphosphonate, DMMP) preconcentrator [23]. The active layers showed good DMMP adsorption capacity when exposed to an atmosphere containing high levels of DMMP (162 mg/m^3^) and a high preconcentration coefficient of 171 when exposed to lower DMMP concentrations (2.6 mg/m^3^), for sample volumes up to 600 STP cm^3^, exceeding the benchmarks of many commercial DMMP adsorbents. Additional measurements were conducted in the presence of water vapor, showing that the material, despite its relative hydrophilicity, does not interfere with the detection of the analyte. Simultaneously, such a measurement result can be considered as proof of the usefulness of the proposed solution in real conditions.

The use of cryogels as materials making up microchannels is a promising solution for the preconcentration and separation of some analytes, such as polycyclic aromatic hydrocarbons (PAHs). In the reported device, two types of cryogels were employed, one acting as a separator and one as a preconcentrator for PAHs and linked directly to a on-column UV-Vis detector [24]. Although some optimisation was required, a good degree of separation was achieved for a mixture of four PAHs (benzo(a)anthracene, chrysene, benzo(a)pyrene, and benzo(b)fluoranthene), with the chromatographic peaks of the individual PAHs being observed at retention times differing by 1–2 min, with the reported limits of detection being on the order of 0.05–0.15 μg/L for each of the four analytes.

**Figure 1:**
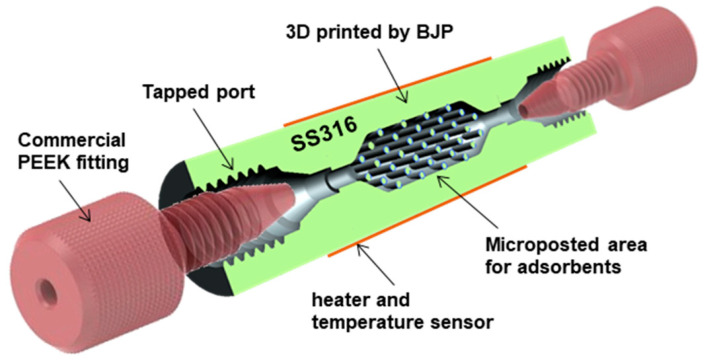
Schematic depiction of the binder-jet printed μPC. Reprinted with permission from [25].

An interesting approach to manufacturing preconcentrators for use in the detection of toluene, as a representative VOC, is to use additive manufacturing methods, such as binder-jet printing (BJP) [25]. The printed preconcentrator (Figure 1) lacked a built-in heater, thus experiments relied on using a heating membrane wrapped around the exterior of the preconcentrator, which resulted in a long period of time being needed for the interior of the preconcentrator achieving the target desorption temperature and necessitating adjustments to the dimensions of the device. Despite this, a comparison of toluene vapour release from the preconcentrator and its direct injection into the sampling channel of the GC-MS instrument showed an underwhelming performance, with a peak area ratio of 14.2 for a sampling volume ratio of 20, showing that this interesting proof of concept device needs significant refinement before being a viable analytical solution.

Another solution for VOC (a mixture of 3-carene, D-limonene, and 1-nonanal in ethanol) preconcentration utilises borosilicate glass wafers, into which the microfluidic channels and adsorbent bed were etched and onto one side of which, a resistive heating and temperature control device was deposited [20]. Although no information about the degree of preconcentration or possible saturation of the adsorbent is given, the preconcentrator design itself was verified to operate correctly and yield highly repeatable concentrations of the thermally desorbed analytes, both across multiple preconcentration-desorption cycles and across individual preconcentrators, as verified by GC measurements.

The first MEMS preconcentrator with miniaturized gas chromatograph/mass spectrometer (GC/MS) detection, which will be sent to the International Space Station in 2021. The PC has a gain of more than 3000, enabling sub-ppm sensitivity of the instrument. The preconcentrator is composed of a silicon heater and a 250-nL chemical trap filled with 80–100-mesh Carboxen 1000 spheres. The GC microcolumn is capable of separating more than 20 VOCs in ppb level sensivity targeted compounds in the air of the ISS cabin [26].

#### 2.1.2. Preconcentration of VOCs for Exhaled Breath Diagnostics

Industry or environmental contamination are not the only sources of VOCs, as some human diseases can lead to the presence of VOCs in exhaled breath [27]. In this case, preconcentration and detection of those compounds becomes a matter of monitoring health. To exemplify, the presence of elevated amounts of acetone in exhaled breath is a sign of either diabetes or starvation [28], whereas elevated levels of e.g., hydrocarbons, alcohols, or ammonia can be used in the diagnostics of multiple afflictions, such as liver impairment and failure [29] or urinary tract infections [30].

In light of the above, the reported micropreconcentrator (μPC), making use of a carbon nanotube foam as the filler for its adsorption chamber [31] is of direct benefit to medical diagnostics. Although the device was found to achieve a greater preconcentration factor for ethane than an analogous one using commercial carbon sieves (respectively, 90.2 and 31.4), it is unclear as to whether this is due to the properties of the nanotubes or merely an effect of an increased specific surface area of the adsorbent, as the two have not been investigated in this aspect.

1-Propanol present in exhaled breath is considered as a biomarker for lung cancer [32], with elevated toluene, o-xylene, and cyclohexane levels being used as auxiliary evidence. The reported μPC and gas chromatography micro-column [33] were fabricated via deep reactive ion etching of silicon wafers, with the cavity of the μPC unit being traditionally equipped with micropillars and having a volume of 20 μL. A commercial DaY zeolite (Degussa) was used as the adsorbent, having the advantages of a large specific surface area (747 m^2^/g, as found experimentally, although it is unclear how the processing of the material affected this parameter) and good physisorption of VOCs. Although information about either the achieved preconcentration factor or the detection limits of the chemoresistive sensor utilised for detecting the analytes were not given, the combined system was found to achieve detection limits of 24 ppb, 5 ppb, 21 ppb, and 112 ppb, respectively for toluene, o-xylene, propanol, and cyclohexane.

**Figure 2:**
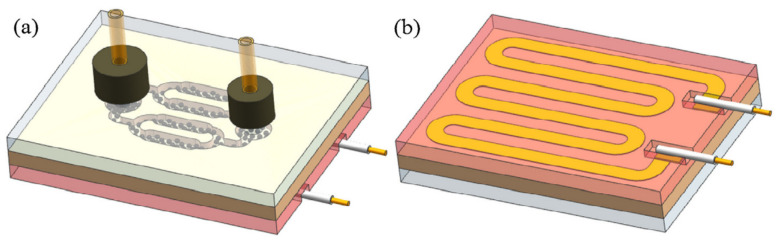
Schematic depiction of the metal gas preconcentrator. (**a**) Structure from the top view, (**b**) the ceramic heater from the backside view. Reprinted with the permission of Elsevier from [34].

In the work of Han, it was proposed to produce μPC that could be used for the non-invasive screening of advanced liver fibrosis by detecting isoprene in exhaled air [34]. The device has been designed as a system of rectangular metal microchannels in the shape of a collector with flat dimensions of 16 mm × 12.6 mm, and an internal void volume of 14.4 μL on a copper substrate. The channels were filled with Carbopack X adsorbent. The scheme of the preconcentrator structure is shown in Figure 2; the detector was a GC-FID device.

Preconcentration factor for 10 ppb of isoprene attains to approximately 352 ppb, and LoDs are determined to be 0.016 ppb. While theoretically it is an analytically useful concentration, the authors, however, did not conduct research in real conditions, and no cross-selectivity studies were carried out, ergo it is difficult to assess the actual usefulness of the proposed solution [34].

Prototype μPC is reported in work [35] for breath sampling and injection in a laboratory gas chromatography coupled with MS. First, a simplification in the injector’s architecture, permitted by the use of a μPC, is shown. The term of sample consumption of the μPC compared to a laboratory TD are demonstrated both on model advantages of synthetic mixtures and breath samples. The signals of three smoking markers in breath, benzene, 2,5-dimethylfuran, and toluene were studied. The μPC, 21 mm × 7.6 mm wafers, were batch processed on 200-mm silicon by standard deep reactive ion etching, as an absorbent porous polymer, based on 2,6-diphenyl-p-phenylene oxide Tenax (TA 60–80 mesh powder), was used. Testing the gas BTEX mix at 500 ppb was sampled. It is important to note that using μPC, 150 mL of breath sample were sufficient to detect the markers. In the study, no LoD was assessed and no calibration was performed. The authors performed the evaluation of the BTEX concentration semi-quantitatively by comparing the peak sizes. It should be added that the degree of pre-concentration was not assessed in the study and the very high sensitivity of the presented μPC was largely attributed to MS detection.

#### 2.1.3. Preconcentrators for Other Species

Au-TiO_2_ nanomaterials have been used in μPCs to detect mercury vapors. The detector of the presented system was a quartz microbalance. The adsorbent material consisted of a layer of titania nanoparticles, decorated with gold nanoparticles. TiO_2_ material was synthesised, starting from titania nanoparticles (anatase phase) suspended in a solution containing HAuCl_4_. Gold nanoparticles were obtained by HAuCl_4_ photo reduction. The pre-concentrator was a single spiral heating pattern, made of Ni-Cr, with a thickness of 150 μm. The system showed an LoD of 5 μg/m^3^, evaluated over a 30-min sampling time. According to these measurements, a sensitivity of 0.034 Hz m^3^/μg min ± 0.003 m^3^/μg min was calculated. The factors interfering with mercury detection were H_2_S and SO_2_ at 90 ppm and 1.12 ppm, respectively [36]. However, the resulting μPC shows a moisture response characteristic of QCM detection. In addition, the lifetime of such a preconcentrator has not been specified, which seems to be particularly important due to hazardous material safety and disposal concerns.

The use of ceramics as micropreconcentrator (μPC) materials is an interesting trend exemplified by a device fabricated from aluminium nitride and intended for the preconcentration of ethylene as a means of monitoring post-harvest fruit spoilage [37]. Although a standard commercial adsorbent, Carbosieve SII (Sigma-Aldrich, Burlington, MA, USA), was used and no information about the preconcentration factor is given, the μPC coupled with a gas chromatograph (GC) achieved a detection limit of 25 ppb for ethylene gas, exceeding the requirements for application in e.g., the food industry and making it a potentially promising solution. The proposed solution appears to have a rather high demand for energy (50 mW). The use of an electrochemical detector allows the miniaturisation of the device, and it not only significantly reduces the price of the entire detection system, but it also does not require qualified personnel to operate it.

Fused silica has been used in a preconcentrator to detect trace amounts of 2,3-dimethyl-2,3-dinitrobutane taggant that is legally required to be added to plastic explosives during their production. The layer of porous silica was formed in situ on the surface of the preconcentrator by etching a boron-doped p-type silicon wafer and then thermally oxidised in oxygen resulting in a porous silica layer on top of a crystalline silicon substrate. The obtained layer was able to concentrate 2,3-dimethyl-2,3-dinitrobutane from 0.5 ppm to 6 ppm, and the GC-MS detection method was used. The advantage of the proposed solution is the low desorption temperature of 70 ${}^{\circ }$C. A nonporous piece of surface-oxidised silicon was used as a control [38].

Another nanosilica-based preconcentrator reported by the authors was found to achieve a three-fold sensitivity enhancement of gas-phase IR detection for nitrobenzene [39].

### 2.2. Preconcentrators Utilising Microextraction and Other Phenomena

An interesting and increasingly popular trend is to employ phenomena other than adsorption to achieve preconcentration. This broadening of methodology has resulted in the development of a variety of μPCs dedicated to liquid-based analytes. Among such μPCs, the use of liquid-liquid microextraction is particularly popular, even though other phenomena, such as electroosmotic flow and electrokinetic trapping are also being explored. Such liquid-phase preconcentrators are already finding application alongside more traditional preconcentration methods [40,41].

An interesting theoretical consideration for the design of micropreconcentrators (μPCs) is to utilise fractal geometries. One such system, dedicated to ionic species and involving a fractal nanochannel, was considered theoretically [42], suggesting that using such novel geometries can increase the analyte enrichment degree.

The use of low-cost and sustainable materials for the fabrication of various devices is a worthwhile trend in materials chemistry. An example of this trend is to replace traditional substrates, such as glass, metals, and silicon, with paper [43]. The microchannels of the reported preconcentrator were produced through a simplistic combination of spraying the paper substrate with a hydrophobic agent and embedding it with paraffin film. This device was equipped with a Nafion membrane, voltage was applied to its ends, and it was treated with 20 μL of a fluorescent tracer solution. The changes in the distribution of this tracer were followed by fluorescence imaging, showing a rapid formation of an ion depletion zone and concentration of the fluorescent tracer on one end of the device, due to the ion concentration polarisation phenomenon, resulting in a reported preconcentration factor of 220 after 40 s of voltage application.

Another electric field-assisted preconcentrator was reported to rely on electroosmotic flow and electrokinetic trapping phenomena, taking place in the vicinity of a Nafion membrane, to achieve in approximately 2 min, a preconcentration factor of approximately 7900 for prostate-specific antigen (PSA) [44]. A particular novelty of this device was that its microfluidic channels were filled with nanobeads functionalised with antibodies capable of interacting with a prostate-specific antigen. The occurrence of such interactions enabled the use of Brownian diffusivity measurements to determine the concentration of the preconcentrated PSA, with a detection limit on the order of 50 pg/mL.

Switchable hydrophilicity solvents have recently become a popular area of research interest due to their ability to alternate between hydrophilic and hydrophobic forms when exposed to e.g., proton donors/proton acceptors. Such a property is promising for many applications, however it is possibly of greatest significance to the field of (micro)extraction [45]. One such switchable solvent, that is octanoic acid, was employed for the purpose of preconcentrating Co^2+^ ions in food and water samples [46]. The developed system involves a 12-step procedure, in which the sample containing Co^2+^ ions, mixed with a complexing reagent (1-nitroso-2-naphthol), is dissolved in a mixture of octanoic acid and an aqueous solution of sodium carbonate. This mixture is then transferred to an extraction chamber and acidified with sulfuric acid, in order to switch octanoic acid to its protonated, hydrophobic form, resulting in phase separation of the system and simultaneous extraction of the analyte. Despite its complexity, the procedure is readily automated, as it relies on the operation of a syringe pump and two multi-position valves, with the entire analytic process taking up almost 11 min. Although the reported enrichment factor of 41 and LoD value of 0.8 μg/dm^3^ for Co^2+^ are not the highest among existing literature reports, the work explores the effect of multiple parameters on the preconcentration process and shows promise as an analytical solution, due to being based on a cost-efficient digital colorimetric detector rather than on a more elaborate system, such as FAAS or ICP-OES.

**Figure 3:**
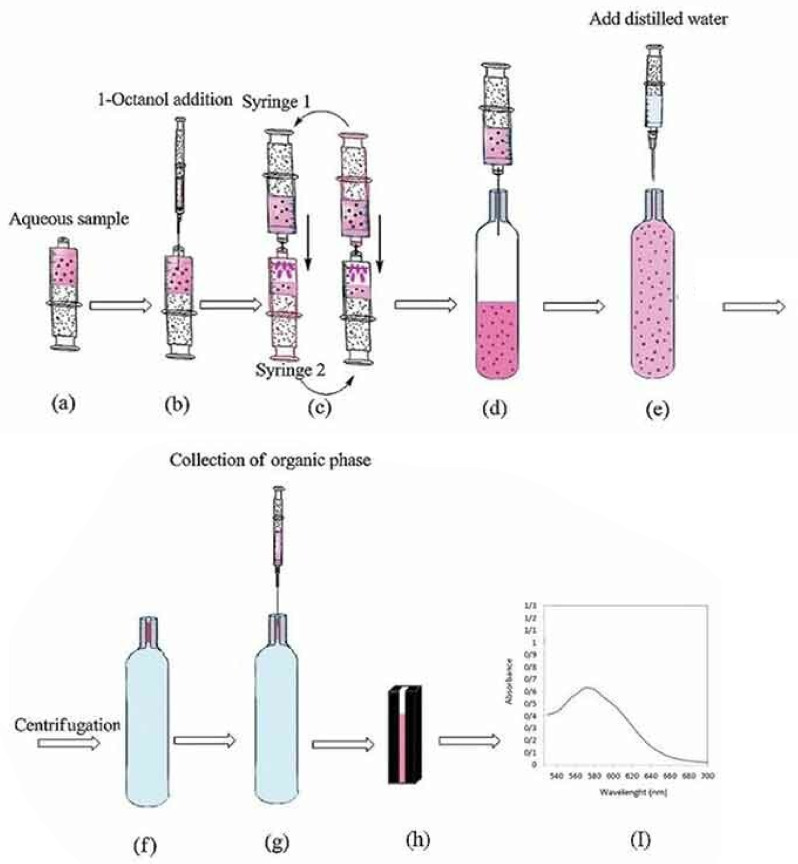
Schematic depiction of a double-syringe liquid microextraction set up for preconcentrating and detecting Hg^2+^ ions. Reprinted with the permission of Taylor and Francis from [47].

The design of μPCs often yields devices that require complex or highly-sophisticated fabrication methods, as seen in some of the works mentioned in this review. Employing liquid-liquid microextraction is an interesting solution (Figure 3) for preconcentration without the use of such fabrication methods, as exemplified in the case of preconcentrating mercury(II) ions [47]. In the reported work, a system of two connected syringes was used as the vessel for microextraction, followed by UV-Vis spectroscopic detection of the analyte in the presence of thio-Michler’s ketone. Despite the straightforward procedure and experimental set up, an enrichment factor of 120 and a LoD of 1.60 μg/dm^3^ was achieved. This is a significant improvement as compared to the previously reported LoD of 9 μg/dm^3^ for the spectroscopic detection of mercury(II) ions in the presence of thio-Michler’s ketone [48].

Liquid-liquid microextraction can be supplemented with further separation methods, such as capillary electrophoresis, for an augmented preconcentration effect, as demonstrated for a series of acidic drugs (warfarin, ibuprofen, naproxen, ketoprofen, and diclofenac) in spiked human capillary blood [49]. Enrichment factors in the range of 29–97 and LoD values in the range of 0.2–3.4 μg/dm^3^ were achieved. Apart from yielding high enrichment factors, the method also allowed rapid sampling processing (7 samples/hour), making it a promising future solution.

## 3. Conclusions

Currently, MEMS technologies are most commonly used for the production of preconcentrators, even though they have a number of disadvantages that hamper the implementation of the developed μPC devices on a wider scale. Those disadvantages include the low mechanical strength of the μPC itself or the need to use cleanrooms. Recent years, however, have brought about a departure from those technologies, with increasing focus being dedicated to such fabrication methods as laser etching, microgenerating, or etching of silica inserts.

Traditionally, for gas and vapour preconcentrators, granular adsorbents are used. In most cases, commercially available adsorbents are employed, although there are some efforts focused on developing new adsorbent materials for μPCs. One significant drawback of granular adsorbents is that their packing causes issues with poor heat transfer and large flow resistance. A better technological solution appears to be the use of adsorbents in the form of thin films or sponges with a developed surface and low flow rates. However, the best concept seems to be to create a μPCs that would combine the functions of a preconcentrator with a chromatographic column and would be able to control analyte desorption as a function of temperature.

A particularly noteworthy development in the subject is the implementation of the phenomena of electroosmotic flow and electrokinetic trapping for the purpose of liquid phase preconcentration. Although such reports have only recently started becoming more popular, this development is extremely significant, as it opens up an entirely new field of application, helping to further improve the sensitivity of liquid-specific detection methods and promote the miniaturisation of those methods as well.

It should be noted that depending on the background of the researchers and on the scope of the experimental work, papers dedicated to the development of μPCs are extremely heterogeneous in terms of reporting the achieved performance benchmarks. Although some variety in this aspect is unavoidable due to practical considerations, either the preconcentration factor or a comparison of the limit of detection (LoD) achieved for a particular analyte detection method with and without the use of the μPC should be reported if the developed device is to be comparable with existing preconcentration and analyte detection solutions. Without a concerted effort in this regard, the development of this crucial field will be hindered by unnecessary ambiguity (Table 1).

## Figures and Tables

**Table 1:** Summary of the preconcentrated analytes and performance achieved by various reviewed μPCs.

Analyte	Detection Method	PC Factor ^a^	Limit of Detection		Refs.
			without PC	with PC	
ethylene	electrochemical ethylene gas sensor (Membrapor)	-	25 ppm	5 ppb	[37]
mercury vapour	QCM sensor ^d^	-	48 ppb ^c^	0.6 ppb ^c^	[36]
gas-phase 2,3-dimethyl-2,3-dinitrobutane	GC–MS	12	-	0.5 ppm	[38]
nitrobenzene	gas-phase IR	-	-	-	[39]
VOCs mixtures	GC-FID ^e^	2300	-	≥13.5 ppb ^c^	[15]
BTEX	GC-PID	-	1–3 ppb	0.057, 0.150, 0.368 ppb	[16]
BTEX mixtures	GC-FID	144	1 ppm	10 ppb	[17]
BTX ^f^	GC-PID ^g^	-	1–3 ppb	20 ppb	[18]
formaldehyde	2D GC-PID	-	2 ppb	0.23 ppb	[19]
VOC mixtures	GC-FID	13.7	-	22 ppb ^b^	[20]
ethanol, acetone, ethyl acetate, benzene	GC-FID	-	200 ppb	2.3, 2.0, 1.3, 0.4 ppb	[22]
DMMP	GC-MS ^h^	171	-	520 ppb	[23]
PAHs	UV–vis	-	-	4.75–19 ppb ^c^	[24]
VOCs	GC-MS	3000	3 ppm	100 ppb	[26]
ethane	GC-FID	90.2		100 ppb	[31]
toluene, o-xylene, propanol, cyclohexane	SnO_2_-based gas sensor	-		24, 5, 21, 112 ppb	[33]
isoprene	GC-FID	352	1.98 ppb	0.016 ppb	[34]
Fluorescent-labeled protein	fluorescence microscope	220	-	-	[43]
Prostate Specific Antigen (P3338, Sigma-Aldrich)	epifluorescence microscope	10,000	1 μg/cm^3^	50 pg/cm^3^	[44]
Co^2+^	colorimetric	41	-	0.8 μg/dm^3^	[46]
Hg^2+^	UV-Vis	120	9 μg/dm^3^	1.6 μg/dm^3^	[47,48]
warfarin, ibuprofen, naproxen, ketoprofen, diclofenac	ESI-MS ^i^	29–97	-	0.2–3.4 μg/dm^3^	[49]

^a^ Preconcentration factor; ^b^ The PC factor was determined for d-limonene only; ^c^ Recalculated to ppb; ^d^ Quartz crystal microbalance; ^e^ Gas chromatography: flame ionisation detector; ^f^ Benzene, toluene, xylene; ^g^ Gas chromatography: plasma ionisation detector; ^h^ Gas chromatography: mass spectrometry; ^i^ Electrospray ionisation mass spectrometry.

## Data Availability

Not applicable.
